# Carbocyanine Dye Usage in Demarcating Boundaries of the Aged Human Red Nucleus

**DOI:** 10.1371/journal.pone.0014430

**Published:** 2010-12-23

**Authors:** Satoru Onodera, T. Philip Hicks

**Affiliations:** 1 Department of Anatomy, School of Medicine, Iwate Medical University, Morioka, Japan; 2 Department of Biology and Faculty of Graduate Studies, Lakehead University, Thunder Bay, Canada; 3 Division of Medical Sciences, Northern Ontario School of Medicine, Thunder Bay, Canada; City of Hope National Medical Center, United States of America

## Abstract

**Background:**

Though the adult human magnocellular Red nucleus (mNr) is essentially vestigial and its boundaries with neighbouring structures have never been well demarcated, human studies *in utero* have shown a well developed semilunar mNr wrapping around the caudal parvicellular Red nucleus (pNr), similar to what is seen in quadrupeds. In the present study, we have sought to better delineate the morphological determinants of the adult human Red nucleus (ahRn).

**Methods and Findings:**

Serial sections of ahRn show fine myelinated fibers arising from pNr and turning toward the central tegmental tract. DiI was deposited within a well restricted region of ahRn at the fasciculus retroflexus level and the extent of label determined. Nissl-stained serial sections allowed production of a 3-D mNr model, showing rudimentary, vestigial morphology compared with its well developed infant homologue. DiI within this vestigial mNr region at the level of the oculomotor nerve showed labeled giant/large mNr neurons, coarse fiber bundles at the ventral tegmental decussation and lateral lemniscal label.

**Conclusions:**

Large amounts of DiI and a long incubation time have proven useful in aged human brain as a marker of long axons and large cell bodies of projecting neurons such as the rubrospinal projection and for clarifying nuclear boundaries of closed nuclei (e.g., the large human pNr). Our 3D model of adult human mNr appeared shrunken in shape and axially rotated compared with the infant mNr, the rotation being a common feature among mammalian mNr.

## Introduction

In adult humans, the magnocellular red nucleus (mNr) which gives rise to the rubrobulbar and rubrospinal tracts, is a relatively vestigal projection [1], [2]. However studies performed with human fetal tissue show that there is a well-developed semilunar mNr which wraps around the caudal extension of the parvicellular red nucleus (pNr) [3], [4], [5], [6]. These two components of the nucleus are well developed and separated from each other. This robust morphology gradually decreases during infancy and early childhood at a time when the pyramidal tract, supported by the rubro-olivo-cerebellar circuit, reciprocally develops.

Using the cat, macaque and human in a recent comparative neuroanatomical study we show that fine myelinated fibers from the human nucleus accessorius medialis of Bechterew (NB) occupy the medial part of the central tegmental tract (CTT) in contrast to the situation in the cat, where the NB occupies an area within the medial tegmental tract (MTT) (Onodera, 1984 [7]). We further demonstrated the possibility of the translocation of the position of the human NB from the ventral central gray to the dorsomedial part of the human pNr (Onodera and Hicks, 2009 [8]). The human ND is isolated in the ventral central gray and occupies the MTT as does the feline ND (in human: see especially Case 1 in Fig. 11.37 Voogd, 2003 [9], in cat: Onodera, 1984 [7], Onodera and Hicks, 1998 [10]). Based on the above observations, we presented the hypothesis of the rolled sheet model of the adult human pNr and mNr, consisting of the rostrolateral extension featuring dorsal and ventral groups of large-giant neurons (Onodera and Hicks, 2009 [8]). There have as of yet, been no clear borderlines of the vestigial human mNr published, likely because the neurons of this region spread only sparsely within the fiber bundle of the superior cerebellar peduncle and adjacent reticular formation [1], [8], [11]. Therefore it is impossible to determine clearly the actual outline of the adult human mNr in contrast to what is seen with the well-developed feline mNr, the latter which represents the so-called typical “closed nucleus”, described by Mannen (1988) [12].

For this study, we applied the definition of “closed nuclei” (Mannen, 1988 [12]) to the human mNr (i.e., the extranuclear dendrites of the mNr cells seldom were found in the neighboring reticular formation) and drew a preliminary outline of the obscure border of the adult human mNr by enclosing the whole area containing all mNr cell bodies and their dendrites, and reconstructed a 3D-model of the red nucleus freehand. We next demonstrated the rubrospinal tract and the CTT through the use of DiI. Generally speaking the use of only very small amounts of DiI crystals in conjunction with young brains permits clear labeling of nerve fibers. For example, we were able to demonstrate clear terminal labeling of the nucleus of Darkschewitsch (ND)-medial accessory olive projection using newborn rat brains and a very small amount of DiI crystal (Onodera et al., 2004 [13]). Although the projecting fibers of aged human brains are not easily suited for DiI experiments, there have been a number of experiments that demonstrated intrinsic local circuitry using aged human brains [14], [15]. Cognizant of the fact that clear labeled projecting fibers using aged human brain tissue are difficult to demonstrate with small amounts of crystal, we ventured to see whether the usage of a relatively larger deposit combined with a very lengthy incubation interval (seven years) would enable us to demonstrate the projecting tracts of the adult human red nucleus.

## Materials and Methods

Human brains were obtained through donations to Iwate Medical University specifically for medical education and research. Corresponding written consents for the usages was given by donors and their families who agreed with policies relating to good–will cadaver donation. Human brain slice preparation, the protocols for which are approved by the Ethics Committee of Iwate Medical University, is used annually in neuroanatomical courses at Iwate Medical University. Cadaver identity data are anonymous. All brain blocking and dye depositions were performed between 1996 and 2003.

### Human Brain Preparation

After death and within 24 h postmortem, cadavers were perfused through the femoral artery with 10,000 ml of a 10% formalin solution. Brains were removed immediately from the skulls and immersed in the same fixative for several months. Well-fixed adult brains (n = 7) were selected and washed in running water. Macro- and microscopical examination of these brains did not reveal any lesions. The diencephalons and brainstems were cut into several blocks. One set of these blocks was cut by a freezing microtome into 50-µm thick serial coronal sections. Each section was picked up with a chrome alum gelatin-coated slide. Every tenth section was stained with 1% cresyl violet. Other serial sections were stained with myelin stain using Luxol fast blue.

Six sets of mesodiencephalic blocks were prepared for carbocyanine dye (DiI, Sigma, 468495) analysis. Three blocks were cut in a coronal plane at the level of the penetration of the fasciculus retroflexus (FR) and three others were cut at the level of penetration of the oculomotor nerve into the Red nucleus. After this cutting, their caudal parts were used for two types of DiI insertion: 1) for demonstration of the CTT, DiI crystal powder (50 to 100 µg per tissue block) was inserted into the dorsomedial and ventrolateral parts of the rostral red nucleus at the level of the FR using the tip of a fine glass pipette (3 cases). 2) For demonstration of the rubro-spinal tract, DiI crystal powder (also 50 to 100 µg per tissue block) was inserted into the ventrolateral and dorsolateral zones of the caudal red nucleus at the level of the oculomotor nerve (3 cases). These DiI-inserted blocks of brainstem were stored in 4% paraformaldehyde in 0.1 M phosphate buffer (PB, pH 7.4) in stoppered glass jars wrapped in aluminum foil and maintained in a dark cabinet at room temperature for seven years. The blocks were then immersed in 0.1 M PB containing 30% sucrose until they sank within the solution. Sections of 100-µm thickness were cut transversely from brainstem blocks on a freezing microtome, collected serially in 0.1 M PB, soaked in a 1∶1 solution of 100% glycerol and 8% paraformaldehyde, and mounted on slides. They were observed and photographed using an epifluorescence microscope (Olympus Vanox) equipped with a standard Rodamine filter set (excitation 520∼550 nm; emission >580 nm).

## Results

### Myelo- and Cytoarchitecture of the Human Parvicellular Red Nucleus

**Figure 1:**
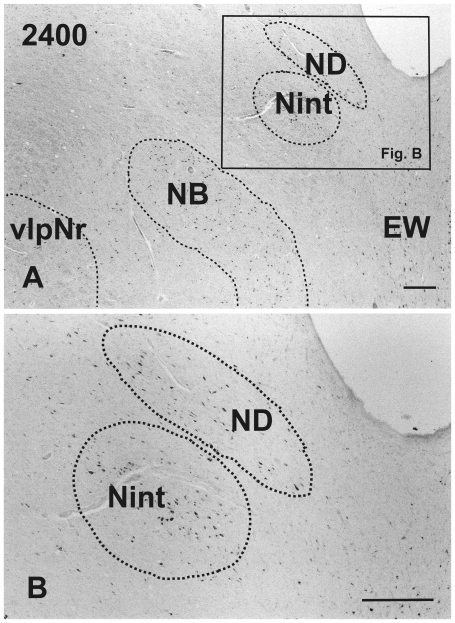
Human ND, Nint and NB. **A:** Photomicrographs showing the distribution of Nissl-stained cells of ND, Nint and NB. B. Isolated ND neurons in the central gray and Nint neurons in the reticular formation. The number in the left corner is the rostrocaudal distance (in micrometers) from the rostral tip of the red nucleus. NB – nucleus accessorius medialis of Bechterew, ND – nucleus of Darkschewitsch, Nint – interstitial nucleus of Cajal. Scale bar  = 200 µm.

**Figure 2:**
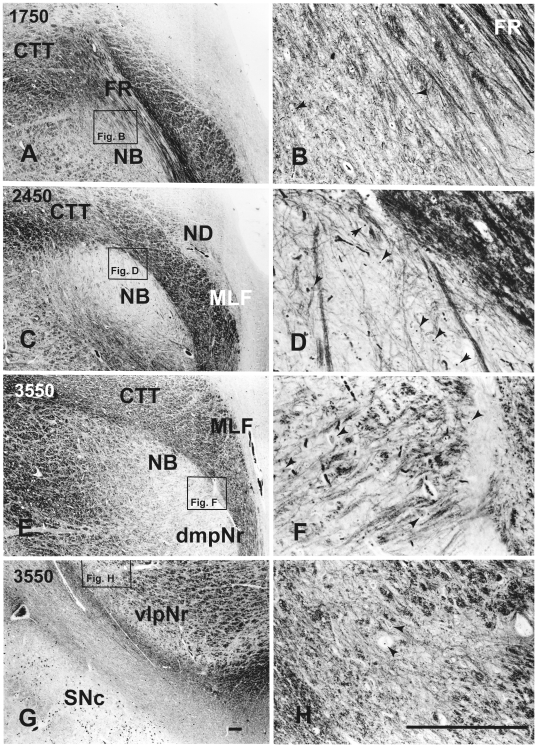
Human parvicellular red nucleus. Photomicrographs showing the distribution of myelinated fibers around NB and its adjacent nuclei (A, C, E and G). B, D, F and H show the distribution of small myelinated fibers. The arrowheads indicate nucleoli of small neurons. CTT – central tegmental tract, dmpNr – dorsomedial part of parvicellular red nucleus, FR – fasciculus retroflexus, mNr – magnocellular red nucleus, NB – nucleus accessorius medialis of Bechterew, ND – nucleus of Darkschewitsch, Nint – interstitial nucleus of Cajal, PBP – parabrachial pigmented nucleus, SNc – substantia nigra pars compacta, vlpNr – ventrolateral part of parvicellular red nucleus. Scale bar  = 200 µm.

Using a series of sections prepared with Nissl (Fig. 1), and a myelin (Fig. 2) stain, we observed the mesodiencephalic nuclei consisting of the ND and the Red nucleus. At the level where the FR passes through the medial side of the NB, the ND is separated from the Red nucleus by fiber bundles consisting of the superior cerebellar peduncle, CTT, MTT and medial longitudinal fasciculus (MLF) (Fig. 1A) and is thus isolated in the ventral central gray (Fig. 1B). Myelin staining of brain sections shows that the FR penetrates and contacts the dorsomedial part of the Red nucleus (Fig. 2A). This well-developed dorsomedial part of the Red nucleus is considered to correspond to the human NB. Furthermore, fine myelinated fibers rise vertically from the NB and join the medial part of the CTT (Fig. 2B, C and D).

At the level of the oculomotor nucleus, oculomotor nerve fibers run along the medial surface of the Red nucleus. Fine efferent fibers from the dorsomedial part of the Red nucleus penetrate the cellular layer and its outer capsule of the fiber bundle, and then descend as the CTT to the inferior olive (Fig. 2 E and F). Fine efferent fibers from the ventrolateral part of the Red nucleus run along the outer capsule, do not penetrate the outer capsule and turn toward the medial direction (Fig. 2G and H).

### Labeling of Human Parvicellular Red Nucleus with DiI

#### Rostral part of the Red nucleus (3 cases)

**Figure 3:**
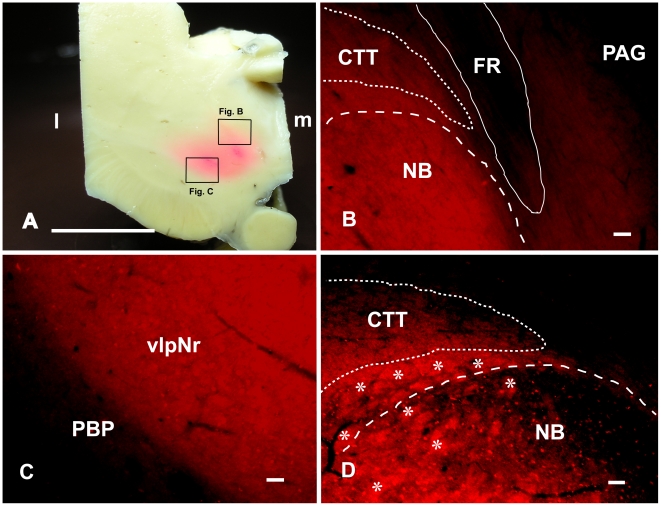
Parvicellular red nucleus and its descending tract. A: Photograph showing points of insertion of DiI crystal into the dorsomedial and ventrolateral parts of the rostral human red nucleus at the level of the fasciculus retroflexus. B–D: Fluorescence photomicrographs showing the distribution of DiI-labeled cell processes consisting of axons, dendrites and glial processes. B: Labeled dorsomedial part (i.e., the NB) of the red nucleus and labeled fiber bundle arising from the NB near the DiI-inserted point. C: Labeled ventrolateral part of the red nucleus near the DiI-inserted point. D: Labeled CTT located over the NB. Asterisk (*) shows labelled fiber bundle of the superior cerebellar peduncle. CTT – central tegmental tract, FR – fasciculus retroflexus, l – lateral, m – medial, NB – nucleus accessorius medialis of Bechterew, SNc – substantia nigra pars compacta, vlpNr – ventorlateral part of parvicellular red nucleus. Scale bar  = 1 cm in A, 100 µm in B–D.

The three cases with DiI showed essentially similar data. In one case, the DiI crystal had spread into the adjacent FR and the surrounding reticular formation. In another case the diffusion of DiI was well-restricted to within the borders of the Red nucleus. Macroscopically and at the sites of DiI crystal insertion, pink-colored, broad, oval areas were observed within the dorsomedial and ventrolateral parts of the Red nucleus (Fig. 3A). Pink-colored extended regions gradually faded into the direction of the fiber bundles of the superior cerebellar peduncle and the CTT (Fig. 3A). Using fluorescence microscopy with rhodamine optics, the boundary area continued from the dorsomedial (Fig. 3B) to ventrolateral (Fig. 3C) parts of the Red nucleus as a strongly labeled zone. At the level of the FR, the NB and its adjacent area were strongly labeled and labeled fiber bundles arose from these areas and ascended dorsally along the FR, although the adjacent FR and periaquaductal gray were unlabeled (Fig. 3B). At a more caudal level, these labeled fine fiber bundles occupied the medial part of the CTT dorsally to the NB (Fig. 3D). In this area of the NB, many small labeled cells were found. The boundary of the ventrolateral part of the labeled Red nucleus was always distinguished from the unlabeled parabrachial pigmental nucleus (Fig. 3C).

These cases show that emerging fibers from NB and its adjacent area occupy the medial part of the CTT. They also introduce the concept that deposition of a relatively large volume of DiI into brain tissue of an aged individual post-mortem, followed by a prolonged time allowed for dye movement is a feasible stratagem for fibre-tracing and nuclear boundary determination.

### Cytoarchitecture of the Human Magnocellular Red Nucleus

**Figure 4:**
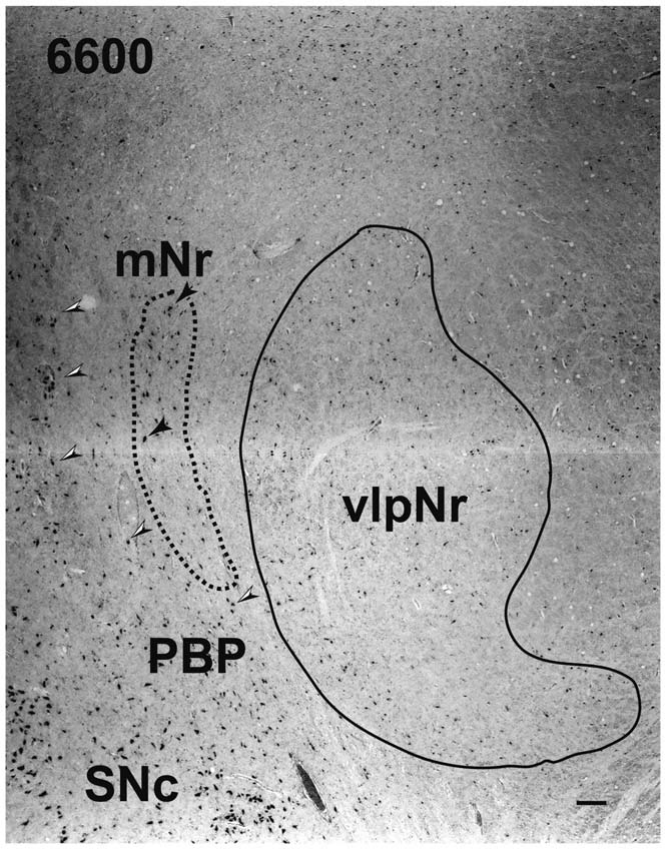
Human magnocellular red nucleus. The area indicated by a broken line is the outer shell of the rostral part of the magnocellular red nucleus outside the parvicellular red nucleus. It lies between the capsule of the superior cerebellar peduncle and the parabrachial pigmented nucleus. The black arrowheads indicate giant neurons. Black and white arrowheads indicate pigmented large neurons. Figs. 4–7, 9 and 10 correspond to rostrocaudally arranged serial sections. Each interval is 600 µm. The number in the left corner is the rostrocaudal distance (in micrometers) from the rostral tip of the red nucleus. mNr – magnocellular red nucleus, PBP – parabrachial pigmented nucleus, SNc – substantia nigra pars compacta, vlpNr – ventrolateral part of parvicellular red nucleus. Scale bar  = 100 µm.

**Figure 5 pone-0014430-g005:**
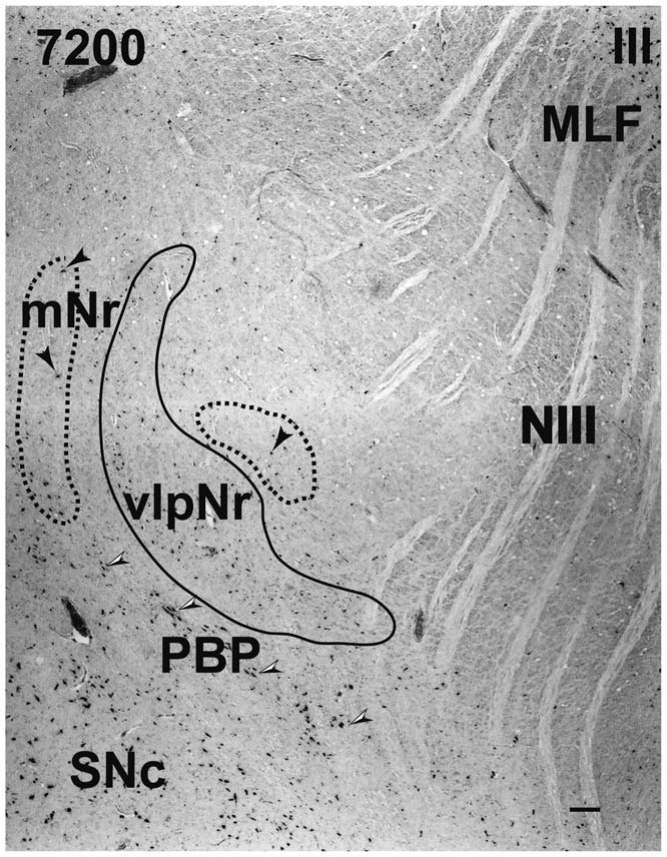
Human magnocellular red nucleus. The two areas indicated by a broken line are parts of the magnocellular red nucleus which appear to pinch the parvicellular red nucleus due to the plane of section. The black arrowheads indicate giant neurons. Black and white arrowheads indicate pigmented large neurons. MLF – medial longitudinal fasciculus, mNr – magnocellular red nucleus, NIII – oculomotor nerve, PBP – parabrachial pigmented nucleus, SNc – substantia nigra pars compacta, vlpNr – ventrolateral part of parvicellular red nucleus, III – oculomotor nucleus. Scale bar  = 100 µm.

**Figure 6 pone-0014430-g006:**
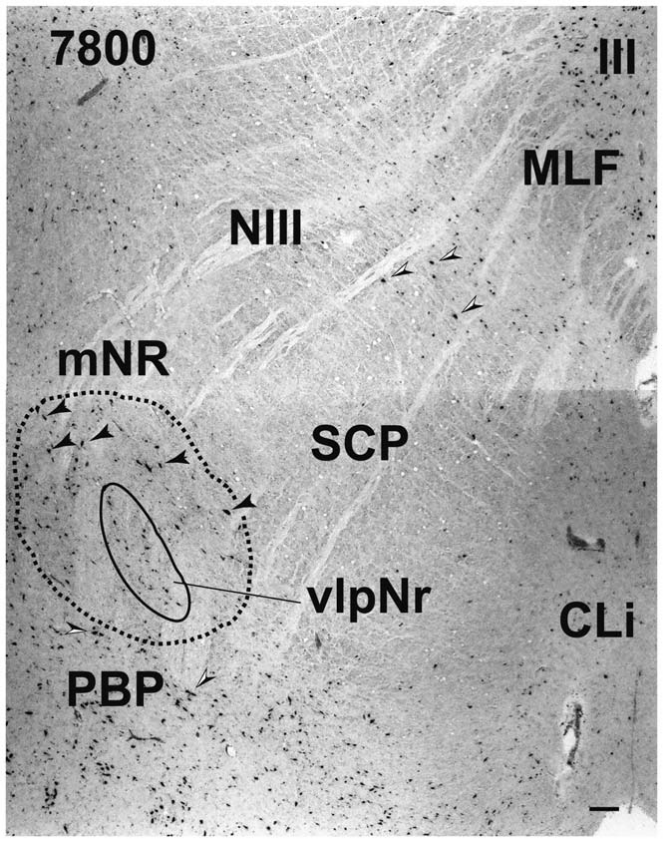
Human magnocellular red nucleus. The magnocellular red nucleus wraps around the caudal pole of the parvicellular red nucleus. The black arrowheads indicate giant neurons. Black and white arrowheads indicate pigmented large neurons. The number in the left corner is the rostrocaudal distance (in micrometers) from the rostral tip of the red nucleus. CLi – caudal linear nucleus, MLF – medial longitudinal fasciculus, mNr – magnocellular red nucleus, NIII – oculomotor nerve, PBP – parabrachial pigmented nucleus, SCP – superior cerebellar peduncle, SNc – substantia nigra pars compacta, vlpNr – ventrolateral part of parvicellular red nucleus, III – oculomotor nucleus. Scale bar  = 100 µm.

**Figure 7:**
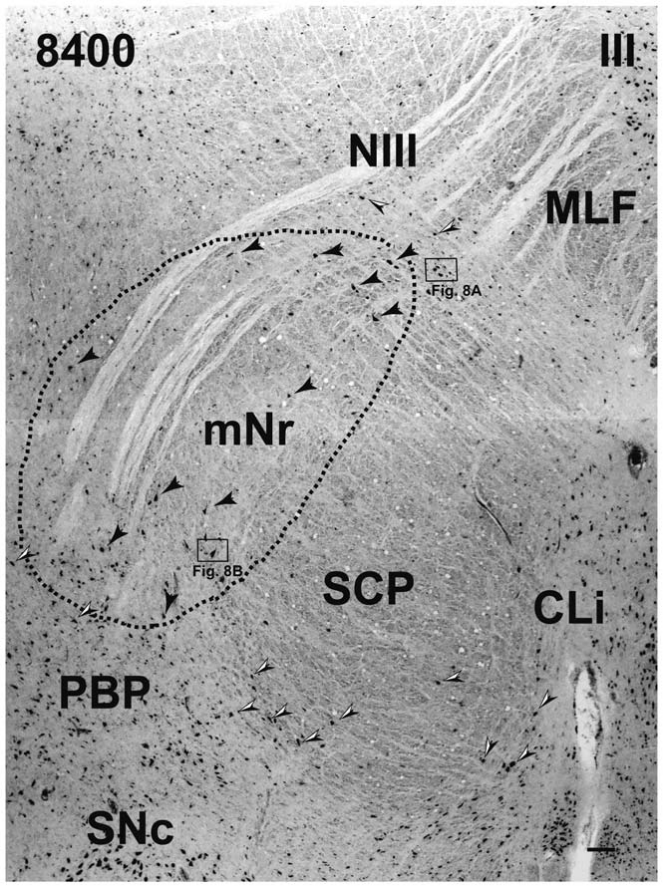
Human magnocellular red nucleus. The largest part of the magnocellular red nucleus. The axis of the mNr rotates dorsomedially. The black arrowheads indicate giant neurons. Black and white arrowheads indicate pigmented large neurons. The number in the left corner is the rostrocaudal distance (in micrometers) from the rostral tip of the red nucleus. CLi – caudal linear nucleus, MLF – medial longitudinal fasciculus, mNr – magnocellular red nucleus, NIII – oculomotor nerve, PBP – parabrachial pigmented nucleus, SCP – superior cerebellar peduncle, SNc – substantia nigra pars compacta, III – oculomotor nucleus. Scale bar  = 100 µm.

**Figure 8:**
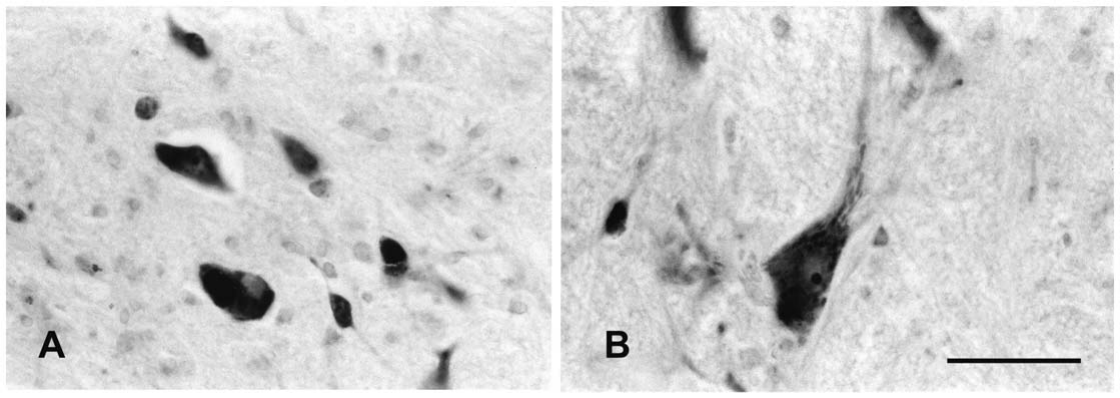
Human magnocellular red nucleus. High magnification photographs of pigmented neurons (A) outside the magnocelluar red nucleus and giant neurons (B) of the magnocellular red nucleus in Fig. 7. Scale bar  = 100 µm.

**Figure 9:**
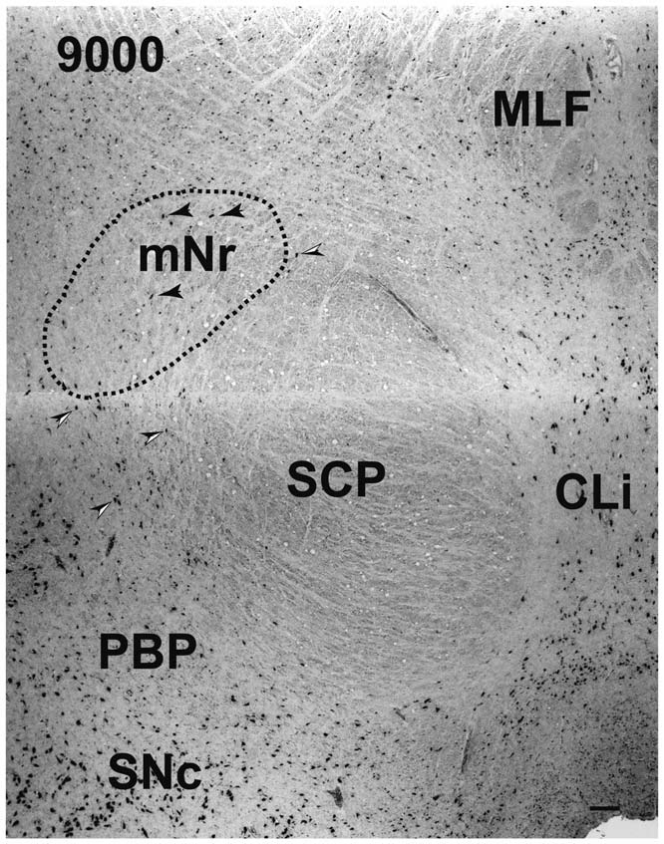
Human magnocellular red nucleus. By this level, the magnocellular red nucleus has been reduced in size. The black arrowheads indicate giant neurons. Black and white arrowheads indicate pigmented large neurons. The number in the left corner is the rostrocaudal distance (in micrometers) from the rostral tip of the red nucleus. CLi – caudal linear nucleus, MLF – medial longitudinal fasciculus, mNr – magnocellular red nucleus, PBP – parabrachial pigmented nucleus, SCP – superior cerebellar peduncle, SNc – substabtia nigra pars compacta. Scale bar  = 100 µm.

**Figure 10:**
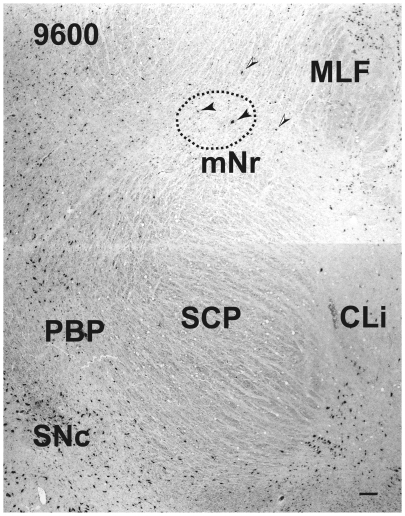
Human magnocellular red nucleus. At this level, the caudal tip of the magnocellular red nucleus has shifted around to its most dorsomedial position. The black arrowheads indicate giant neurons. Black and white arrowheads indicate pigmented large neurons. The number in the left corner is the rostrocaudal distance (in micrometers) from the rostral tip of the red nucleus. CLi – caudal linear nucleus, MLF – medial longitudinal fasciculus, mNr – magnocellular red nucleus, PBP – parabrachial pigmented nucleus, SCP – superior cerebellar peduncle, SNc – substantia nigra pars compacta. Scale bar  = 100 µm.

At the caudal level of the vlpNr, large, medium and small sized neurons were seen to be gathered between the outer capsule of vlpNr and the pigmented (see next paragraph) cell layer (Figs. 4 and 5). This neural zone and scattered giant and large neurons of the mNr form the semi-lunar shell. Many large, pigmented neurons are observed in the surrounding area outside the Red nucleus and superior cerebellar peduncle, such as the parabrachial pigmental nucleus and the rostral and caudal linear nuclei. The caudal pole of pNr appears to be "wrapped" by scattered mNr neurons (Fig. 6); these form an oval area surrounding the caudal pole region. The long axis of this oval area is inclined in a dorsolateral-ventromedial direction. The long axis of the widest area of the mNr turns toward the dorsomedial-ventorlateral direction (Fig. 7). In this area, giant neurons as well as large neurons of the mNr are gathered largely into a dorsal and a ventral group and are scattered among the fibers of the superior cerebellar peduncle. There is the possibility that some large pigmented neurons near the Red nucleus would be mis-identified as mNr neurons (Fig. 7). However, these neurons can be clearly eliminated as non-mNr neurons since the cell bodies of non-rubral neurons contain neuromelanin pigment (Fig. 8A) while mNr cells do not (Fig. 8B). The latter contain coarse Nissl bodies (Fig. 8B). The size of the mNr gradually decreases as one moves caudally (Fig. 9). The most caudal pole of the mNr shifts dorsomedially (Fig. 10)

### A 3D-Model of the Magnocellular Red Nucleus

**Figure 11:**
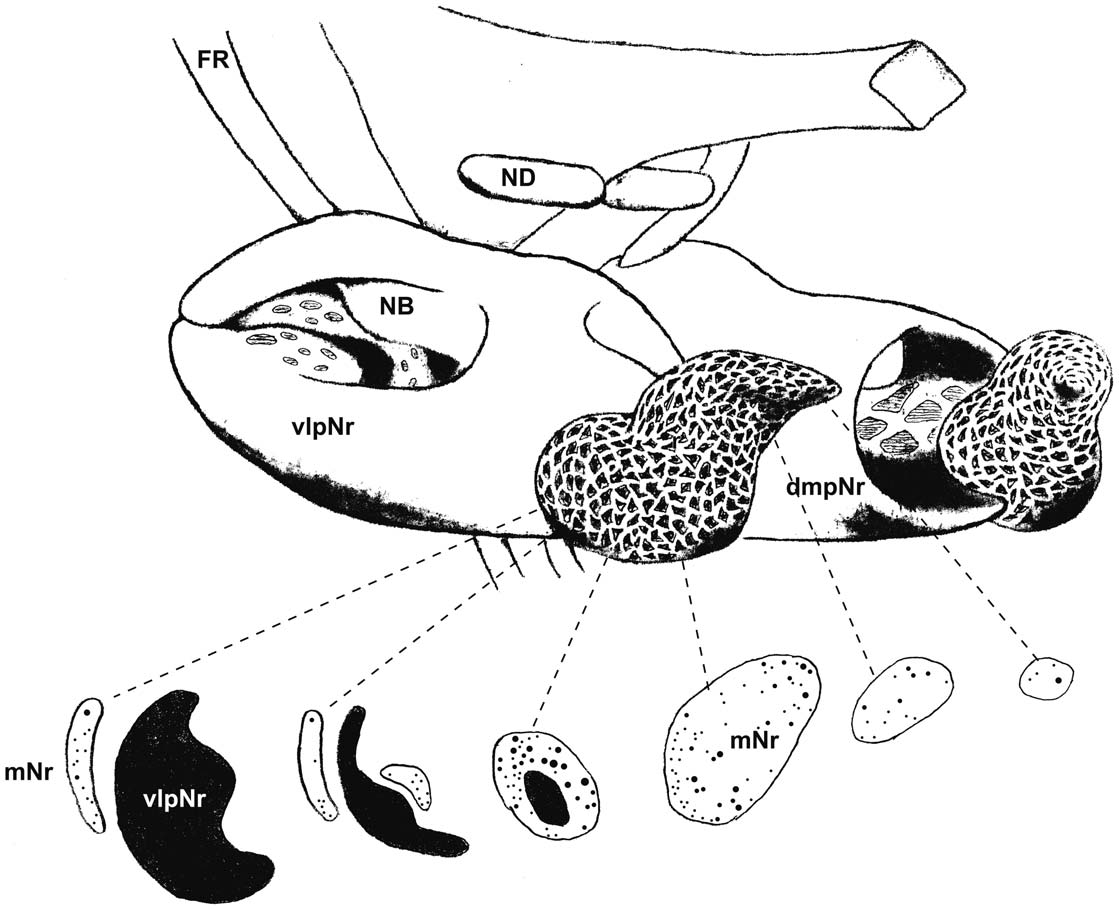
Three-dimensional model of human red nucleus. Upper drawing shows a three-dimensional model of the human red nucleus. The rostral end of this model is toward the left side. The lower drawing shows serial sections of the magnocellular red nucleus (also shown in Figs. 4–9) for reconstructing the magnocellular red nucleus. Large dots indicate giant neurons. Top extension with diamond-shaped cross-section is the cerebral aqueduct in the mid-saggital plane, dmpN – dorsomedial part of parvicellular red nucleus, FR – fasciculus retroflexus, mNr – magnocellular red nucleus, NB – nucleus accessorius medialis of Bechterew, ND – nucleus of Darkschewitsch, vlpNr – ventrolateral part of parvicellular red nucleus.

A 3D-model (Fig. 11) of the mNr was drawn using serial sections of the Nissl stained tissue (Figs. 4–7, 9 and 10). The caudal tails of the mNr are directed in the dorsomedial direction (Fig. 11 shows both right and left mNrs). The surface of the mNr appears as a very coarse weave, containing giant neurons as well as large neurons that are scattered among the fibers of the superior cerebellar peduncle. By contrast the surface of the pNr is formed by a very dense presence of small neurons that are covered by a capsule comprising the fibers of the superior cerebellar peduncle.

### Labeling of the Human Magnocellular Red Nucleus with DiI

#### Caudal part of the Red nucleus (3 cases)

**Figure 12:**
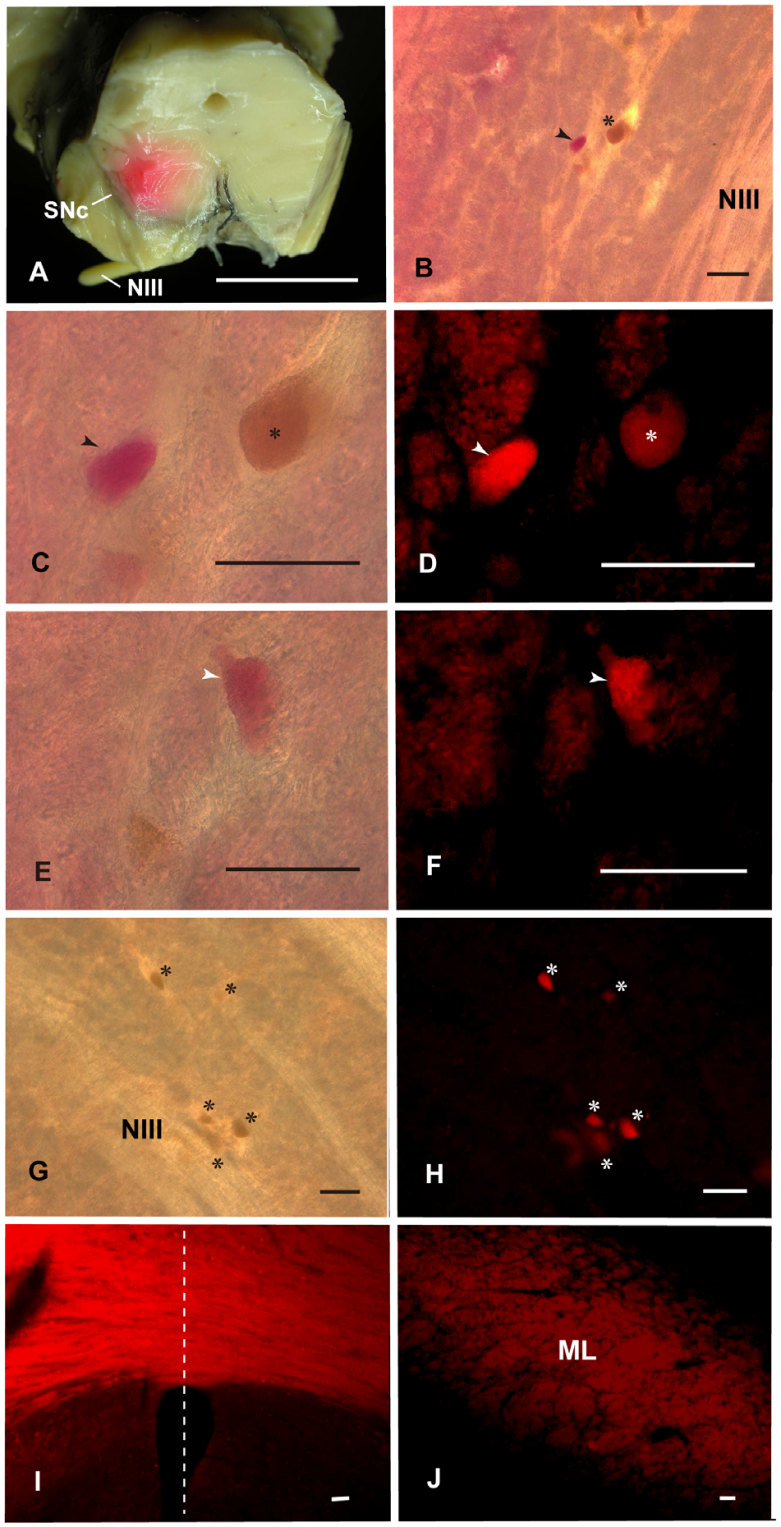
Caudal red nucleus and its descending tract. A: Photograph showing points of insertion of DiI crystal into the dorsolateral and ventrolateral parts of the caudal human red nucleus. B: Low magnification, bright-field photomicrograph of the ventrolateral part of the caudal red nucleus. Arrowhead shows pink-colored, large neuron. Asterisk (*) shows brown-colored, large neuron which possesses lipofuscin granules. C and E: High magnification of pink-coloured, large cells. D and F: Fluorescence photomicrographs of C and E. G and H: Contralateral mNr cells. I: Labeled coarse fibers at the ventral tegmental decussation: i.e., the decussating rubrobulbar and rubrospinal fibers. The dashed line indicates the midline. J: Labeled medial lemniscus ipsilateral side. ML – medial lemniscus, NIII – oculomotor nerve. Scale bar  = 1 cm in A, 100 µm in B–J.

The three DiI cases also showed essentially similar results. The DiI inserted was seen to spread beyond the border of the Red nucleus and into the surrounding structures. In all cases, the ipsilateral medial lemniscus (ML) could be traced from the level of the inserted point to the level of over one centimeter caudally. Under macroscopic observation and at the site of the DiI crystal insertion, a pink-colored, broad circular area was observed (Fig. 12A). This labeled area expanded into the surrounding reticular formation, superior cerebellar peduncles and adjacent ML. Under bright-field microscopy, pink-colored, giant neurons and large neurons were observed near the oculomotor nerve fibers at the ventrolateral and dorsomedial region of the caudal Red nucleus (Fig. 12B and C) and these pink-labeled neurons demonstrated stronger fluorescence than the neighboring autofluorescent large neurons which possessed brownish lipofuscin granules (see Fig. 12C-F). Contralaterally, large neurons possessing lipofuscin granules also showed autofluorescence (Fig. 12G and H). Large neurons containing neuromelanin pigment did not autofluoresce in the surrounding area outside the Red nucleus. The coarse fibers of the ventral tegmental decussation were labeled at the level of the oculomotor nucleus (Fig. 12I) and contained fibers of the decussating rubrobulbar and rubrospinal tracts. The labeled ML was observed at the section and was separated by over one centimeter caudally from the DiI-inserted point (Fig. 12J).

These cases demonstrate that there are labeled, giant and large mNr neurons that have coarse decussating labeled fibers running through the rubrobulbar and rubrospinal tracts.

## Discussion

### Structure of Adult Human Red Nucleus

#### Parvicellular and Magnocellular Red Nucleus

Recently we showed that the parvicellular part of the adult human Red nucleus consisted of a zonal, sheet-like structure similar to that in the cat and monkey and we proposed further that the human nucleus exists as a rolled sheet: essentially proposing a novel model for the human pNr structure (Onodera, 1984 [7], Onodera and Hicks, 2009 [8]). We offered the view that in humans, the nucleus accessorius medialis of Bechterew is not an integral part of the ventral central gray, like it is in the cat and monkey, but that it corresponds to the dorsomedial part of the Red nucleus (Onodera and Hicks, 2009 [8]). By this translocation of the human NB, fine myelinated fibers from the human NB occupy the medial part of the CTT (as demonstrated by the present DiI study) in contrast to the situation in felines, where the NB occupies an area within the MTT (Onodera, 1984 [7]). The human nucleus of Darkschewitsch is separated from the nucleus accessorius medialis of Bechterew, and is isolated in the ventral central gray as was shown in Fig. 1.

In the present study the borders of the vestigial human mNr were based on the criteria of Mannen's “closed nucleus” as established by Golgi staining in cats: i.e., the extranuclear dendrites of the mNr cells were seldom found in the neighboring reticular formation [12]. Such criteria correspond well for other species, e.g., for mouse mNr neurons intracellularly labeled with neurobiotin (Jiang et al. 2002 [16]).

Miller and Strominger (1973 [17]) showed in the monkey that neurons with a coarse Nissl pattern (i.e., mNr neurons) extend for a short distance along the lateral side of fine Nissl pattern neurons (i.e., pNr neurons) in the rostral part of the Red nucleus. We are of the view that this short dorsolateral extension of the monkey's mNr corresponds to the rostral extension of human mNr (Figs. 4 and 5, see also Onodera and Hicks, 2009 [8]). At the level of the oculomoter nerve passing through the superior cerebellar peduncle, the largest area of the mNr contains the ventral and dorsal groups of giant and large cells and its long axis dorsomedially inclined (Fig. 7). The caudal tip of the mNr is located in the dorsomedial area of the midbrain reticular formation (Fig. 9) and this dorsomedial point of the caudal mNr corresponds to the position of the mNr as drawn in “Atlas of Human Brainstem” [18].

Our 3D-model of the human mNr shows a very coarse surface and shrunken shape, compared with the fetal semilunar swollen shape, its axis rotating from the dorsolateral-ventromedial direction to the dorsomedial-ventrolateral direction (Fig. 11). This rotation is a common feature among the mNr of various mammalian species, although there is a developmental difference among species within the mammalian mNr. Padel et al., (1981) [19] showed that the gibbon mNr is laminated and is rotated about its major axis. This feature seems closely compatible with our suggested model for the human mNr.

Rubrospinal fibers are of large diameter and are relatively sparse. We demonstrated labeled decussating rubrospinal fibers (Fig. 12I) in our material. However, rubroolivary fibers are very thin and densely packed (*cf*. the central tegmental tract) (Onodera, Hicks, 2009 [8]). Therefore we were not able to demonstrate individual isolated fibers of the rubroolivary tract using the large amount of DiI crystal in our study; however it was notable that fusion bundles of rubroolivary fibers were labeled (Fig. 3B and D).

### The third component of the Red Nucleus

Data from rats [20], cats [21], [22], [23], [24] and monkeys [25], [26] have demonstrated that a dense cluster of small cells lying dorso-lateral to the rostral pole of the mNr represents a third component of the Red nucleus; it is considered as being separate because this region is cytoarchitecturally segregated from the original mNr and pNr and it has different neural connections from the mNr and pNr. These small, scattered cells which receive input from the cerebellar lateral nucleus [24] project to the contralateral spinal cord via the rubrospinal tract and lack any input from motor cortex [25]. This is in contrast to the situation in the mNr, where there are inputs both from the cerebellar anterior interpositus nucleus and the motor cortex. This mesencephalic reticular formation area that contains the dense cluster of small cells just described, receives input from the superior colliculus (the mNr does not); functionally this area relates to horizontal saccadic eye movement [27]. Therefore this third component and the mNr serve different roles for the control of body movements. The third component may relate to eye, facial, neck, trunk and proximal limb movements and the mNr to jaw and distal limb movements [24], [25], [27], [28]. Since the cerebellar lateral nucleus [24] but not the motor cortex [25] provides input to this third component, it may be related functionally to quick responses, such as ocular reflexes involved in protection, rather than voluntary jaw and limb movements. This same third component may also exist in the human Red nucleus. Papez and Stottler (1940) [29] showed that in human infants, a group of small cells located caudal and lateral in the Red nucleus project to the contralateral spinal cord.

The present study was not concerned with this third component that projects to the spinal cord, since this component and the mNr have different connections and roles, as described above.

### Phylogeny and Ontogeny of the Human Red Nucleus

In humans, the red nucleus consists of the pNr and it derives the CTT, whereas the cat's red nucleus contains the mNr, the origin of the rubrospinal tract. In a pioneering study of this region in cats, Ogawa (1939) proposed that the feline pNr corresponded to a nuclear complex consisting of the nucleus of ND, the interstitial nucleus of Cajal and the nucleus of the fields of Forel, forming the MTT [30]. Onodera (1984) [7] showed the cat's homolog region of the human pNr to consist of the NB and pNr, and demonstrated further that the NB projects to the ventral lamella of the principal olive via the MTT while the pNr projects to the dorsal lamella of the principal olive via the CTT. Recently we proposed the rolled-sheet model of the human pNr: in this proposal, the human (as in the cat) ND occupies the MTT, while the human NB translocates to the dorsomedial part of pNr, along the phylogenetic scale of the apes. That is, the chimpanzee as well as the human exhibit a complete separation between ND and its well-developed red nucleus, whereas the gibbon shows only an hourglass-shaped space between the ND and the NB [31]. The human NB occupies not the MTT but the medial part of the CTT [8]. This model is positively supported by human brain materials (Voogd, 2003) [9] and experimental animal data from chimpanzee (Strominger et al., 1985 [32] and macaque (Strominger et al., 1979 [33]).

Based on observations made from human fetal tissue, Yamaguchi and Goto (2008) [6] showed that there was a complex feature of the rostral half of the pNr. They observed a deep groove in the fasciculus retroflexus on its medial surface and a short longitudinal fissure on its dorsolateral surface. The caudal part has a relatively smooth and ovoid appearance where the neurons are preferentially distributed near the outer margin, while they are relatively scarce in the central region. These features of the fetal pNr are in good agreement with and support our rolled sheet model of the human adult pNr (Onodera and Hicks, 2009 [8]).

### Possible Functional Implications

mNr neurons in quadrupeds (cat; [34]) exhibit a significant increase in discharge frequency during the movement of either fore- or hindlimbs over obstacles, similar to what is seen with pyramidal tract neurons. Animals executing voluntary gait modification of fore- and hindlimbs use visual and other sensory cues [35]. Pyramidal tract neurons contribute to fine, precise control of distal limb movements both by increases and decreases in the level of spinal interneuron activity. By contrast, mNr neurons may discharge more in relation to intralimb coordination as well as interlimb coordination [35]. mNr neurons of rats and monkeys, species that possess a manipulable hand, are provided with a “hand preshape” through their extension of digits via goal-directed limb movements, such as reaching to grasp (Whishaw and Gorny 1996 [36], van Kan and McCurd 2001, 2002 [37], [38]). Pyramidal tract neurons superimpose motor control for individual finger movements on grouped-finger extension, such action which is governed by rubrospinal tract neuron activity. In rats having mNr lesions, very rapid grasping behaviour and an absence of a pause in limb transport during grasping occurs (Whishaw and Gorny 1996 [36]). In monkeys pyramidotomy leads the changes in mNr organization, such as loss of normal extensor performance, that contribute to the recovery of weakness of the flexor muscle of the forelimb. This suggests that there is a branching of axon terminals from extensor rubro-motoneuronal cells to flexor rubro-motoneuronal cells (Belhaj-Saif and Cheney 2000 [39]).

We can conclude from these and other observations that these dual complementary control systems provide a continuously active template, or framework, of movement programming upon which the pyramidal system adds motor program overlays to produce relatively more fractionated movements (Whishaw and Gorny 1996 [36], Belhaj-Saif and Cheney 2000 [39]). Animals having such long, descending pyramidal tracts were able to maintain a more stable condition of their body posture and therefore were able to execute more precise skilled movements of their hands [40].

The macaque and baboon [17], species which are proficient at terrestrial quadrupedalism, exhibit a very well-developed mNr. However, the mNr of apes (e.g., gibbon and chimpanzee) shows a consistent trend to decrease gradually along the phylogenetic scale [19], [41], [42], [43]. This might be the reason why ape and human newborns are not able to turn over from a back-lying position, although newborn monkeys are easily able to perform such movements. The complication of spontaneous general movement of four limbs from a back-lying position is no different between upper and lower limbs in chimpanzee babies, whereas in human two-month olds the complication of general movement of the lower limbs from the back-lying position is reduced to a more simple pattern, e.g., reciprocal kicking, than is the complication of general movement of the upper limbs [44]. These differences suggest that the chimpanzee mNr was retained to provide patterns of neural activity supporting four hands-preshape for reaching to grasp, while the shrinking human mNr lost the foot preshape for reaching to grasp, while the developing human pyramidal tract developed to provide a pattern of activity supporting foot programs for bipedal purposes. The chimpanzee's mNr is the smallest and most limited in size for quadrupedalism and arboreal life; the foot of the chimpanzee has retained its hand-like grasping ability via control through both rubrospinal and pyramidal tract systems. We may therefore be justified in assuming that the actual size of the early hominin's mNr occupied a position somewhere between that of the chimpanzee and present-day humans. Anatomical data (Sobel 1977 [45]) have shown that the cell count in the chimpanzee's mNr (*ca*. 1,250) is 2.8 times greater than that in humans (*ca*. 450 cells). Neurons of the human mNr are found in a variety of sizes: giant, large, medium and small cells (Sobel 1977 [45]). Humans are known to possess 150–200 giant-to-large sized neurons in that portion of the mNr which project large myelinated fibers not only to the brain stem but also within the first three cervical segments [1], [2].

Other studies concerning the human fetal mNr have shown that this structure develops progressively during the latter half of gestation and that the mNr is more prominent in fetal stages than in adulthood (Ulfing and Chan 2001, 2002, [3], [4] Yamaguchi and Goto, 2006 [5]). The fetal mNr drapes around the caudal third of the pNr in the form of a semi-lunar shell, and it and the pNr are clearly separated from each other. This morphological situation is transitory. This diminution of the mNr would have resulted in an organism having an increased accuracy of skilled corporeal movement and may have led to the first steps of bipedalism through a functional, or operational transition from motor commands routed through the rubrospinal tract to routing through the pyramidal tract (Onodera and Hicks, 1999 [46]). Observation of human infants makes evident that once certain key milestones are achieved for stabilization of the various body segments (head, upper and lower torso segments, crawling behavior), standing is achieved and balancing behavior performed, as is low-velocity, bipedal locomotion that typically begins around one year of age. The acquisition of upright bipedalism demands not only the maturation of the body's phenotype but also the reorganization of the nervous system (Sarnat, 2003 [47]).

Although axonal growth cones of pyramidal tract neurons reach only to the lowest aspect of the sacral cord by mid-gestation, the maturation of the pyramidal tract is not complete until around two years of age, as defined by two important criteria: 1) myelination, and 2) sprouting of terminal axonal collaterals to make multiple synaptic contacts on both motoneurons and interneurons of the spinal ventral horns (Sarnat, 2003 [47]). These maturational events begin after 30 weeks of gestation. Therefore the well-developed rubrospinal tract is believed to have a more important neurological function in fetus, neonate and infant than it does in the adult, which has the rubrospinal tract function supplanted by the stronger, or more dominant influence of the pyramidal tract.
